# PINK1/Parkin-Mediated Mitophagy Regulation by Reactive Oxygen Species Alleviates Rocaglamide A-Induced Apoptosis in Pancreatic Cancer Cells

**DOI:** 10.3389/fphar.2019.00968

**Published:** 2019-09-03

**Authors:** Chunle Zhao, Ruizhi He, Ming Shen, Feng Zhu, Min Wang, Yuhui Liu, Hua Chen, Xu Li, Renyi Qin

**Affiliations:** Laboratory of Biliary-Pancreatic Surgery, Department of Biliary-Pancreatic Surgery, Affiliated Tongji Hospital, Tongji Medical College, Huazhong University of Science and Technology, Wuhan, China

**Keywords:** rocaglamide A, mitophagy, apoptosis, reactive oxygen species, pancreatic cancer

## Abstract

Pancreatic cancer (PC) is one of the most lethal diseases, and effective treatment of PC patients remains an enormous challenge. Rocaglamide A (Roc-A), a bioactive molecule extracted from the plant *Aglaia elliptifolia*, has aroused considerable attention as a therapeutic choice for numerous cancer treatments. Nevertheless, the effects and underlying mechanism of Roc-A in PC are still poorly understood. Here, we found that Roc-A inhibited growth and stimulated apoptosis by induction of mitochondria dysfunction in PC. Moreover, Roc-A accelerated autophagosome synthesis and triggered mitophagy involving the PTEN-induced putative kinase 1 (PINK1)/Parkin signal pathway. We also demonstrated that inhibition of autophagy/mitophagy can sensitize PC cells to Roc-A. Finally, Roc-A treatment results in an obvious accumulation of reactive oxygen species (ROS), and pretreatment of cells with the reactive oxygen species scavenger *N*-acetylcysteine reversed the apoptosis and autophagy/mitophagy induced by Roc-A. Together, our results elucidate the potential mechanisms of action of Roc-A. Our findings indicate Roc-A as a potential therapeutic agent against PC and suggest that combination inhibition of autophagy/mitophagy may be a promising therapeutic strategy in PC.

## Introduction

Pancreatic cancer (PC) is a lethal disease. Curative resection is the first to consider for early-stage patients, but 5-year survival is only up to 25% (Kamisawa et al., 2016, Deplanque and Demartines, 2017). For most patients with advanced PC, chemotherapy, such as gemcitabine and oxaliplatin, is the mainstay of treatment. However, the majority of PC is often resistant to chemotherapies (Deplanque and Demartines, 2017; Seufferlein and Ettrich, 2019). Therefore, new therapeutic measurements for PC are urgently needed.

Macroautophagy (hereafter referred to as autophagy) is an evolutionarily ancient and highly conserved cellular degradation process that involves the formation of double-membraned vesicles called autophagosomes that engulf defective proteins and organelles for delivery and digestion to the lysosome. Autophagy is involved in multiple biological activities and functions to maintain cellular homeostasis. Recent studies showed that autophagy plays dual roles in tumor promotion and suppression in variable cancers depending on different cell backgrounds (Levy et al., 2017; Hao et al., 2019). Autophagy can be activated in response to a number of stressors including cancer chemotherapeutics, facilitating cell survival and leading to treatment resistance. However, autophagy is overactivated in certain conditions and ultimately causes cell death. Thus, the importance of autophagy in tumor progression and drug response has led to a focus on autophagy in cancer treatment (Amaravadi et al., 2016).

Mitophagy is a specific type of autophagy in which impaired mitochondria are specially targeted for degradation to promote mitochondrial turnover and maintain cellular homeostasis (Drake et al., 2017). The PTEN-induced putative kinase 1 (PINK1) and Parkin-mediated pathway is widely accepted as a main mechanism of mitophagy (McWilliams and Muqit, 2017). When the mitochondrial membrane potential (쉙*Δψm*) is disrupted, PINK1 transiently stabilizes on the outer membrane of damaged mitochondria and forms a large complex on the outer membrane surface, where it recruits Parkin to impaired mitochondria (Clark et al., 2006; Villa et al., 2017). Parkin ubiquitinates various substrates and promotes clearance of the damaged mitochondria. Abnormal mitophagy was associated with diverse pathologies, and loss of either PINK1 or Parkin leads to accumulation of damaged mitochondria (Villa et al., 2017). Some studies have shown that mitophagy is frequently inactivated in cancer, but the mechanism is not fully understood (Lleonart et al., 2017). Furthermore, several reports showed that mitophagy is involved in tumor resistance to various cancer therapies by removing damaged mitochondria and maintaining healthy and functional mitochondria (Pickles et al., 2018). Therefore, clarifying the underlying mechanism of mitophagy is crucial for exploiting tumor therapeutic target.

Rocaglamide A (Roc-A) belongs to a group of phytochemicals that are isolated from the tree *Aglaia elliptifolia* (Ebada et al., 2011). Roc-A exhibits potent anticancer activities and can inhibit tumor growth at nanomolar concentrations through multiple mechanisms in several cancer models (Li-Weber, 2015; Wu et al., 2017, Yao et al., 2018). However, the mechanism of Roc-A in PC has not been fully elucidated. In our study, we clarified the action of Roc-A in PC cells. Our data demonstrated that Roc-A provokes PC cell apoptosis by induction of mitochondria dysfunction while activating PINK1/Parkin-mediated protective mitophagy. These findings demonstrate a novel relationship between Roc-A and mitophagy, which is of particular guidance for a potential strategy for PC treatment.

## Materials and Methods

### Chemicals

Anti-LC3B (No. ab51520), anti-caspase-3 (No. ab32351), and FUNDC1 (No. ab74834) were purchased from Abcam; anti-PARP (No. 9532) was purchased from Cell Signaling Technology (Beverly, MA, USA); anti-GAPDH (No. 60004-1-Ig), anti-Bax (No. 50599-2-Ig), anti-Bcl-2 (No. 12789-1-AP), anti-Bcl-xl (No. 26967-1-AP), anti-cytochrome *c* (No. 66264-1-Ig), anti-PINK1 (No. 23274-1-AP), anti-Parkin (No. 14060-1-AP), and anti-BINP3L (No. 12986-1-AP) were purchased from Proteintech Group (Chicago, IL, USA). Goat anti-rabbit and rabbit anti-mouse secondary antibodies were purchased from Boster (Wuhan, China). Rocaglamide A (Roc-A; No. HY-19356, purity: 96.43%), necrosulfonamide (No. HY-100573, purity: 99.23%), ferrostatin-1 (No. HY-100579, purity: 99.72%), and Mdivi-1 (HY-15886, purity: 98.75%) were obtained from MedChemExpress (Monmouth, NJ, USA). Z-VAD-FMK was purchased from Millipore (Bedford, MA, USA). Chloroquine (CQ; No. C6628), bafilomycin A1 (No. B1793), and *N*-acetyl-l-cysteine (NAC; No. A7250) were obtained from Sigma-Aldrich (St. Louis, USA). The chemicals were handled according to suppliers’ instructions and treated at the required working concentrations.

### Cell Culture

All cells (PANC-1, MIA PaCa2, TKF-1, HepG2, HCT-116, LO2, and HK-2) were purchased from American Type Culture Collection (Manassas, USA). PANC-1 and MIA PaCa2 cells maintained in Dulbecco’s modified Eagle medium (DMEM) (PANC-1, MIA PaCa2, HepG2, HCT-116, and LO2) or RPMI-1640 medium (TFK-1) containing 10% fetal bovine serum (FBS), 1,000 units/L of penicillin and 1,000 mg/l of streptomycin under humidified 5% CO_2_ atmosphere at 37°C.

### Cell Viability Analysis

Cell counting Kit-8 (CCK-8) assay (Boster, Wuhan) is used to evaluate cell viability. Cells were seeded into the 96-well plate (3 × 10^3^ per well). After 24 h, cells were treated with chemical agents as indicated in figures. Water-soluble tetrazolium salt (10 μl) was added to each well, and cells were incubated for 1.5 h at 37°C with 5% CO_2_, and absorbance was measured at 450 nm by microplate reader (Thermo Scientific, China). The average percentage of viable cells at each group was computed as follows: Survival Rate % = (OD treated − OD blank)/(OD control − OD blank) × 100%.

### Colony Formation

Cells are seeded in a 6-cm dish (2 × 10^3^ per dish), treated with dimethyl sulfoxide (DMSO) or Roc-A for 12 h, and replaced with fresh medium, which was maintained for 2 weeks. Then colonies were photographed after fixation with 4% paraformaldehyde for 0.5 h and 0.1% crystal violet for 1 h. Experiments were run independently in triplicate.

### Xenograft Experiments

Animal experiments were approved by the Committee on Ethics of Animal Experiments of Huazhong University of Science and Technology and performed in accordance with the Association for Assessment and Accreditation of Laboratory Animal Care guidelines. Nude BALB/c mice at 6 to 8 weeks old were obtained from HFK BioTechnology (Beijing, China). Mice were injected subcutaneously with 100 μl ofserum-free DMEM containing PANC-1 cells (2 × 10^6^ cells). When the tumors reached 200–250 mm^3^, mice were randomly divided into two groups (*n* = 5): One was injected with Roc-A (4 mg/kg of body weight), and the other was injected with 1% DMSO in olive oil (100 μl) through the abdominal cavity once every 3 days.

### Apoptosis Detection

Annexin V-FITC/PI Apoptosis Detection Kit (Beyotime Biotechnology, China) was used to detect cell apoptosis according to the manufacturer’s instructions. Briefly, cells were treated with indicated treatments, collected, and resuspended in 1× binding buffer with annexin V-FITC (2.5 μl per well) and propidium iodide (PI; 5 μl per well) and incubated in the dark for about 15 min, and then cell suspension was analyzed by flow cytometry.

### Western Blotting Analysis

For total cellular protein isolation, cells were harvested and lysed on ice in RIPA buffer (Boster Biological Technology, Wuhan, China). For cytosolic fraction isolation, we used mitochondria/cytosol fractionation kit (Beyotime, China) according to the manufacturer’s instruction. After centrifugation at 12,000 rpm for 15 min, the supernatant was collected. Protein concentration was measured by the bicinchoninic acid protein assay kit (Beyotime, Haimen, China). Protein with equivalent amounts from every sample was resolved on sodium dodecyl sulfate–polyacrylamide gel electrophoresis (SDS-PAGE) gel, transferred onto a poly(vinylidene diﬂuoride) (PVDF) membranes (Millipore, MA, USA), and blocked with 5% fat-free milk; the corresponding primary antibodies were incubated at 4°C overnight; the bands using TBST solution were washed; the indicated secondary antibodies (Boster, Wuhan, China) were incubated; and bands with ChemiDoc^™^ XRS+ with Image Lab^™^ Software (Bio-Rad, USA) were detected and analyzed.

### Oxygen Consumption Rates

The oxygen consumption rate (OCR) was measured using an XF96 Extracellular Flux Analyzer (Agilent Technologies, Santa Clara, CA). Briefly, PC cells were treated in the presence and absence of Roc-A (50 nM) for 24 h and seeded at an appropriate density in an XF96 plate. Cells were cultured for 24 h at 37°C in 5% CO_2_. Then cells were incubated for an additional 1 h in a non-CO_2_ incubator in media that were replaced with seahorse basal medium supplemented with 25 mM of glucose and 4 mM of l-glutamine. The oxygen consumption rate was monitored at basal conditions and after sequential injection of the mitochondrial modulators oligomycin (1 µM), carbonyl cyanide *p*-(trifluoromethoxy)phenylhydrazone (FCCP, 2 µM, Millipore Sigma No. C2920), and antimycin A (10 µM, Millipore Sigma No. A8674). The oxygen consumption rate data will be changed to protein levels, the non-mitochondrial respiration was subtracted from all measurements, and antimycin A was added.

### Cytoplasmic Calcium Levels

The PC cells grown to 75% were treated with Roc-A for 24 h. The collected cells were rinsed three times with phosphate-buffered saline and stained with 3 μM of Fluo-3/AM (Beyotime, S1056) in an incubator for 30 min. Then cells were rinsed with phosphate-buffered saline three times, incubated for another 20 min, and analyzed by flow cytometry.

### Confocal Laser Scanning Microscopy (CLSM)

Cells were transfected with GFP-LC3B or GFP-mRFP-LC3B and seeded on glass coverslips in six-well plates. According to the designated measurements, cells were fixed with 4% formaldehyde for 0.5 h. To detect whether autophagosome and mitochondria were co-located, we used GFP-LC3 and MitoTracker to separately mark autophagosome and mitochondria. Cells transfected with GFP-LC3B were seeded on glass coverslips and treated with indicated treatments, stained with MitoTracker, and fixed with 4% paraformaldehyde for 10 min. Cells were photographed using a confocal microscope LSM710 (Carl Zeiss, Oberkochen, Germany).

### Transmission Electron Microscopy (TEM)

After cells were treated with indicated treatments, cells were fixed in 2.5% glutaraldehyde for 1 h at 4°C and post-fixed with 1% osmium tetroxide for 1 h at 4°C, and cells were embedded in spur resin. Images were captured by TEM (Hitachi, H-7000FA).

### Mitochondrial Transmembrane Potential (MMP, Δψm) Assessment

After cells were incubated with Roc-A according to the indicated times and concentrations, cells were collected and incubated with a fluorescent dye JC-1 working solution (5 μg/ml) for 20 min at 37°C, centrifuged, and washed twice. MMP was assessed by detecting the fluorescence of separated cells using FACSCalibur flow cytometer (Becton Dickinson, USA).

### Transient Transfection and RNA Interference

Lentiviral vectors harboring GFP-LC3B or GFP-mRFP-LC3B reporter were purchased from GenePharma (Shanghai, China). The specific small interfering RNA targeting ATG5 (5′-GUGAGAUAUGGUUUGAAUA-3′), Parkin (5′-​GGATCAGCAGAGCATTGTTCA-3′), and PINK1 (5′-GGACGCTGTTCCTCGTTAT-3′), and the corresponding control was purchased from RiboBio (Guangzhou, China). Cells were transfected with Lipofectamine 2000 (Invitrogen) according to the manufacturer’s instructions. Forty-eight hours after transfection, the efficiency of transfection was assessed by western blotting.

### Detection of ROS

The fluorescent dye 2,7-dichlorofluorescein diacetate (DCFH-DA) (Beyotime, China) was used to measure production of intracellular reactive oxygen species (ROS). Cells were treated with indicated treatments, collected, stained with 10 μM of DCFH-DA, resuspended, incubated in serum-free DMEM with 10 μM of DCFH-DA for 0.5 h, removed from DMEM, and washed with PBS twice. FACSCalibur flow cytometer (Becton Dickinson, USA) was used to detect generation of the fluorescence and to assess ROS generation.

### Statistical Analyses

All statistical analyses were performed using the GraphPad Prism 7 software (GraphPad, La Jolla, CA, USA). The quantitative data are presented as the means ± SD and means ± SEM. Statistical differences were evaluated by using Student’s t-test, and statistical significance was assessed by using one-way analysis of variance (ANOVA) followed by Tukey’s post-hoc test at *p* < 0.05 (*p < 0.05; **p < 0.01; ***p < 0.001).

## Result

### Roc-A Inhibits Growth of PC Cells *In Vivo* and *In Vitro*

**Figure 1:**
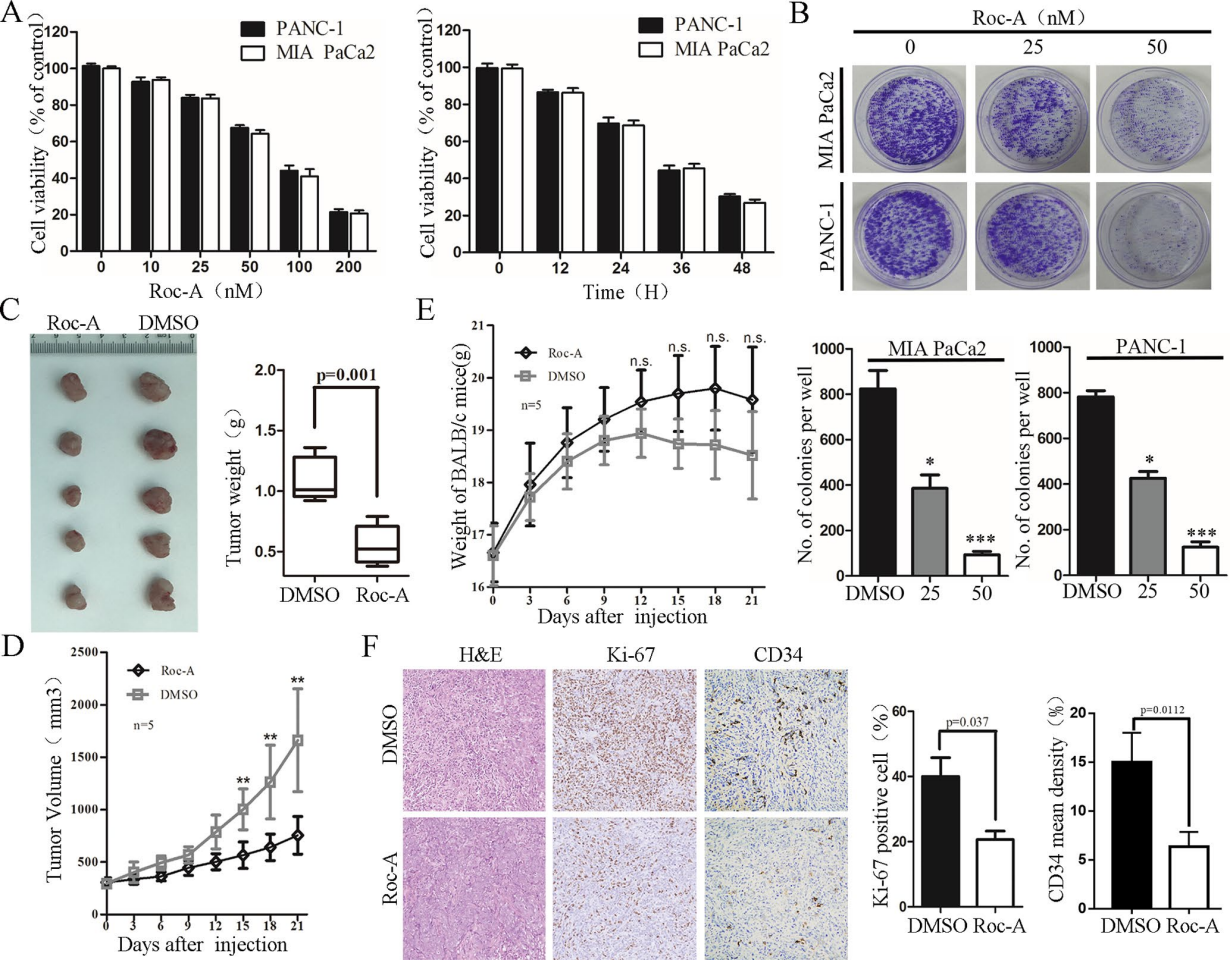
Roc-A inhibits growth of PC cells *in vivo and in vitro*. **(A)** MIA PaCa2 and PANC-1 cells were treated with Roc-A at increasing concentrations for 24 h and increasing duration at 50 nM; cell viability was measured by CCK-8. Data are presented as mean ± SD from three independent experiments. **(B)** Representative images from colony-formation assay. Each bar presents mean ± SD from three independent experiments. The asterisks indicate a statistically significant effect of treatment. *p < 0. 05; **p < 0.01; ***p < 0.001. **(C)** Tumors of subcutaneous mouse xenograft were shown from two groups. **(D)** Tumor volume was presented when tumor volume reaches 200–250 mm^3^. Tumor volume was measured once every 3 days. Data are presented as mean ± SEM from three independent experiments. **p < 0.01. **(E)** Mouse weight was presented when tumor volume reaches 200–250 mm^3^. Mouse weight was measured once every 3 days. **p < 0.01. F. Images of hematoxylin and eosin (H&E), Ki-67 (p=0.037), and CD34 staining (p=0.0112) were shown in tumor tissues from two groups.

We first examined the effect of Roc-A on PC cells growth by CCK-8 assay. We treated MIA PaCa2 and PANC-1 with 50 nM of Roc-A at different times or with different concentrations of Roc-A for 24 h. The results showed that Roc-A inhibited PC cell viability in time- and dose-dependent manner (**Figure 1A**). Colony-formation assay further indicated that Roc-A could inhibit the long-term proliferation in PC cells (**Figure 1B**). Meanwhile, we examined the effect of Roc-A on other gastrointestinal tumor cell growth and primary noncancerous cell lines by CCK-8 assay. As indicated in **Supplement 1**, the majority of gastrointestinal tumor cells are sensitive to Roc-A including TFK-1 (human cholangiocarcinoma cells), HepG2 (human hepatoma carcinoma cells), and HCT-116 (human colon cancer cells). In contrast, LO2 (human live cells) and HK-2 (human renal tubular epithelial cells) are not sensitive to Roc-A. To examine the antitumor effects of Roc-A *in vivo*, we constructed a subcutaneous mouse xenograft model generated by implanting PANC-1 cells. The Roc-A-treated group showed a significant reduction of tumor weight and tumor volume than did controls (**Figures 1C, D**). Notably, no difference in the weight of mice between the two groups was observed (**Figure 1E**). We performed immunohistochemistry on paraffin-embedded xenograft tumors and found that Ki-67 proliferation marker and tumor vascularization index (CD34) showed a lower expression in the Roc-A group than did the vehicle group (**Figure 1F**). Together, these results demonstrate that Roc-A inhibits PC cell proliferation* in vitro* and *in vivo*.

### Roc-A Induces PC Cell Apoptosis

**Figure 2:**
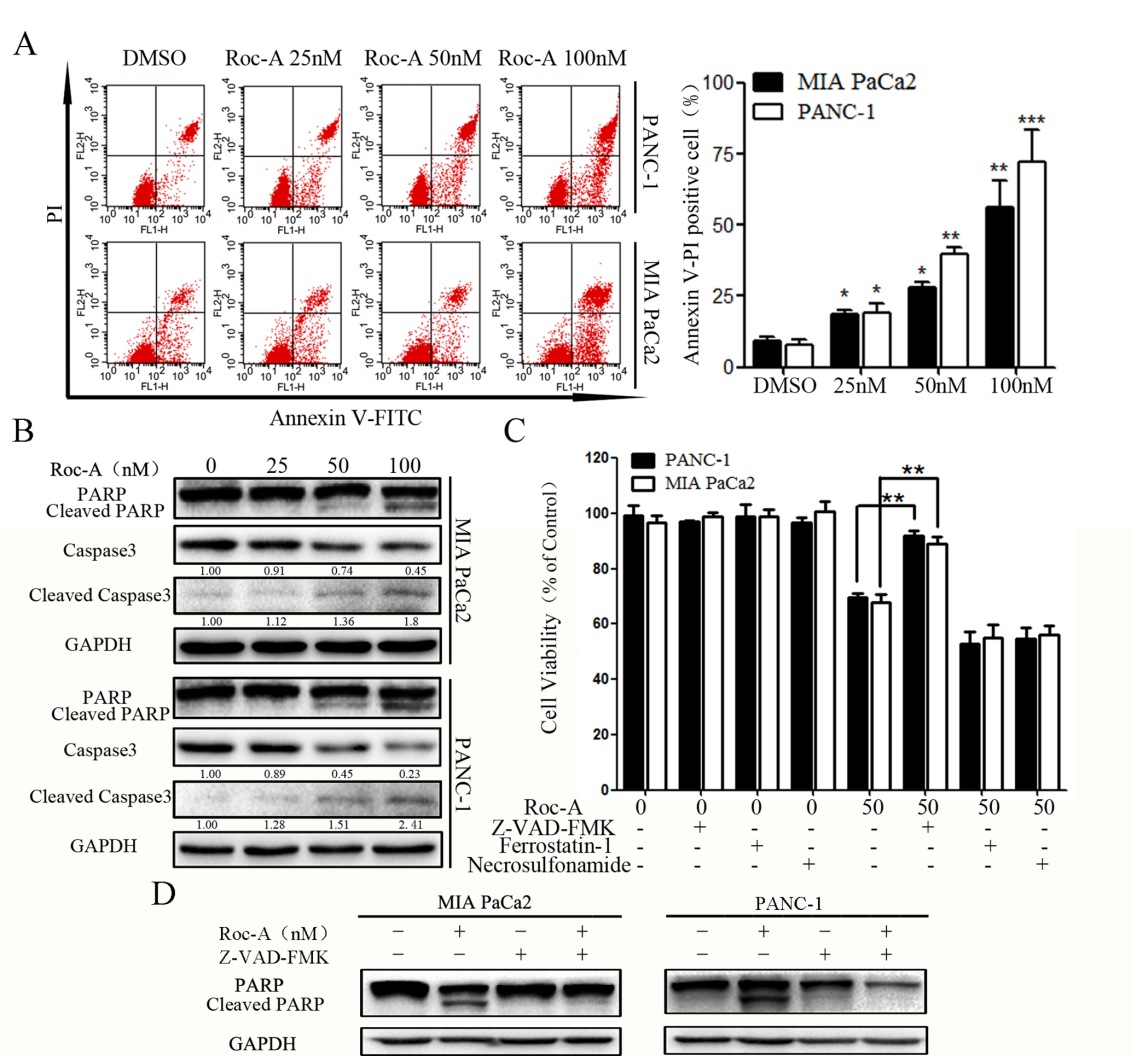
Roc-A induces PC cells apoptosis. **(A)** Flow cytometry detected apoptotic cells after cells were treated with indicated concentration. *p < 0.05; **p < 0.01; ***p < 0.001. **(B)** Western blotting detected caspase-3 and PARP levels. Cells were treated with Roc-A for 24 h, and the supernatant for western blotting was extracted. **(C)** Cells were incubated with Roc-A (50 nM) and Z-VAD-FMK (50 µM) or ferrostatin-1 (20 nM) or necrosulfonamide (20 nM) for 24 h, and cell viability was measured. Data are represented as the means ± SD (*n* = 3); *p < 0.05; **p < 0.01. **(D)** Western blotting analysis of PARP levels. Cells were incubated with Roc-A (100 nM) and Z-VAD-FMK (50 µM) for 24 h.

To determine whether the cell growth inhibitory effect of Roc-A was associated with cell apoptosis, we examined the apoptotic ratio of PC cells using annexin V/PI staining and flow cytometry. As shown in **Figure 2A**, the apoptosis rate of PC cells was gradually increased in a concentration-dependent manner. To explore the mechanisms of Roc-A-induced PC cell death, we examined the activation of caspase-3 and PARP by western blotting. The level of cleaved caspase-3 and cleaved PARP increased in a dose-dependent manner in response to Roc-A treatment (**Figure 2B**). However, levels of GPX4 (a ferroptosis related index), caspase-1, and cleaved caspase-1 (pyroptosis-related index) showed no changes in Roc-A-treated cells compared with controls (**Supplement 2**). To clarify the mechanism of Roc-A-mediated induction of cell death, we used Z-VAD-FMK (a pan-caspase inhibitor), ferrostatin-1 (a ferroptosis inhibitor), and necrosulfonamide (a necroptosis inhibitor) to examine the Roc-A-mediated effects on cell viability in CCK-8 assays. We found that Z-VAD-FMK partially prevented Roc-A-mediated PC cell death, whereas ferrostatin-1 and necrosulfonamide had no obvious effects (**Figure 2C**). Western blotting further demonstrated that Z-VAD-FMK decreased the level of cleaved PARP under Roc-A treatment (**Figure 2D**). Together, our results demonstrate that Roc-A induces PC cell death involved in a caspase-dependent apoptotic pathway.

### Roc-A Induces Mitochondrial Dysfunction and Activates the Mitochondrial Apoptosis Pathway in PC Cells

**Figure 3:**
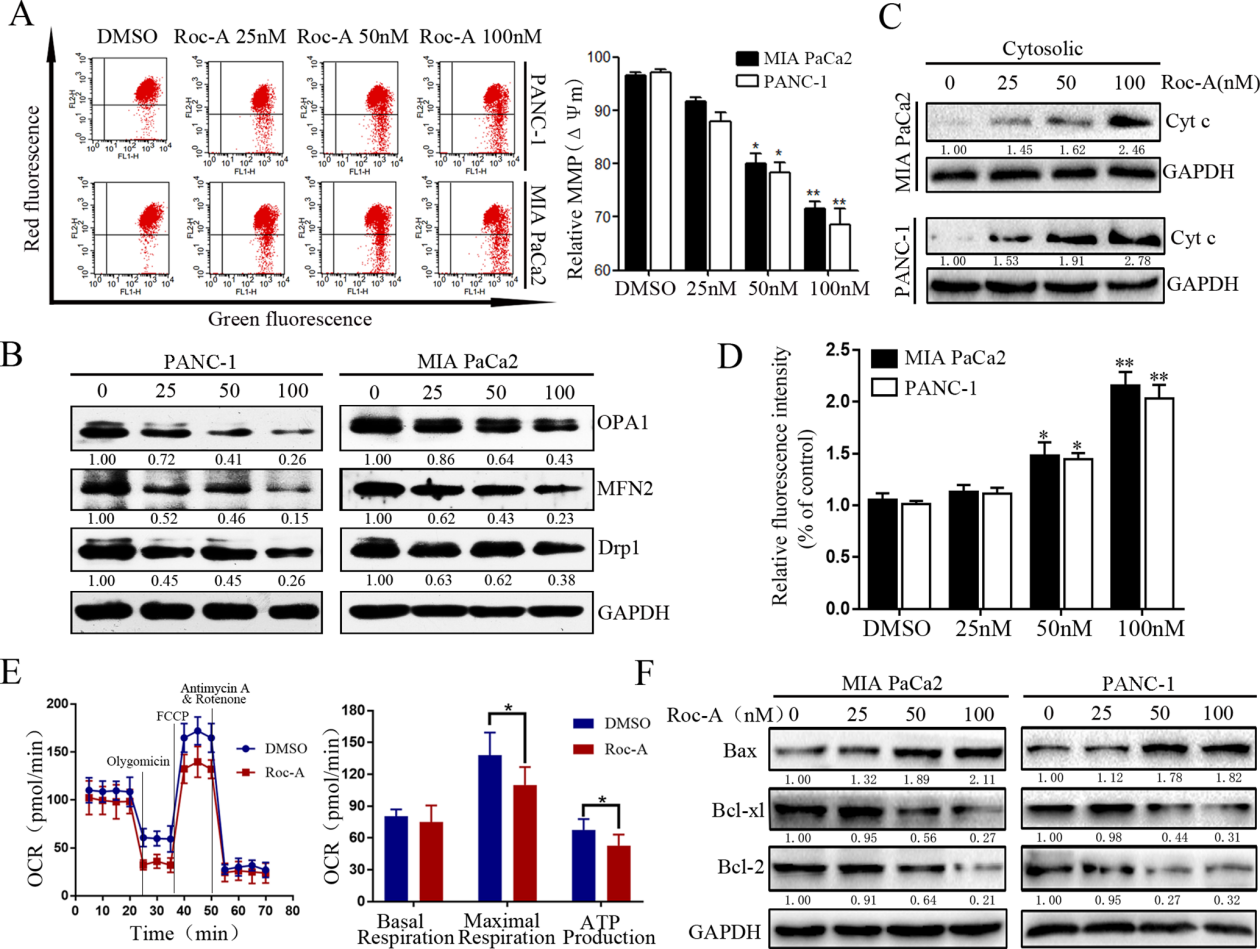
Roc-A induces mitochondrial dysfunction and activates mitochondrial apoptotic pathway in PC cells. **(A)** MMP (쉙*Δψm*) was detected after cells were treated with Roc-A in indicated concentration for 24 h. Data are presented as means ± SD from at least three independent experiments (*p < 0.05; **p < 0.01). **(B)** Western blotting analysis of Drp1, OPA1, and MFN2 expressions. **(C)**. Western blotting detected the release of cytochrome *c* from mitochondria to cytosol. **(D)** The concentration of cytoplasmic calcium in Roc-A-treated cells was analyzed. Data depicted in the graph are expressed as percentages based on the control. The asterisks indicate a statistically significant effect of treatment (*p < 0.05; **p < 0.01). **(E)**. Cells were treated with 50nM of Roc-A for 24 h and measured with the seahorse analyzer. OCR was measured continuously throughout the experiment at baseline and in the presence of the indicated drugs. (*p < 0.05). **(F)** Western blotting analyzed the mitochondrial apoptosis-related proteins.

Mitochondria play an important role in regulating cell physiology and survival. The *Δψm* is an important index that reflects the functional status of the mitochondrion, and its depletion results in the initiation of apoptotic cascades (Zhang et al., 2015). To evaluate whether Roc-A-induced PC cell apoptosis is related to mitochondrial dysfunction, *Δψm* and the expression of mitochondrial fission/fusion proteins were assessed. We found that *Δψm* significantly decreased in a dose-dependent manner under Roc-A treatment (**Figure 3A**). Moreover, Roc-A markedly decreased the expression of mitochondrial fission proteins (Drp1) and the fusion proteins (Mfn2 and OPA1) (**Figure 3B**). Previous studies have reported that reduction of *Δψm* can induce the release of apoptosis factors from mitochondria to the cytosol, such as cytochrome *c* (Kroemer et al., 2007). We next evaluated the level of cytochrome *c* in the cytosol and found that cytochrome *c* levels were increased in the cytosol after Roc-A treatment (**Figure 3C**). Next, we measured further the energetic status of PC cells upon Roc-A administration by evaluating cytoplasmic calcium levels, oxygen consumption rates, and ATP levels. As shown in **Figures 3D, E**, increasing concentrations of Roc-A increased cytosolic-free calcium levels and reduced oxygen consumption rate and ATP production. Moreover, we measured the expression of Bcl-2 family proteins by western blotting and found that Roc-A treatment markedly increased the protein levels of Bax and decreased the protein levels of Bcl-2 and Bcl-XL (**Figure 3F**). Together, these data demonstrate that Roc-A induces mitochondrial dysfunction involved in a mitochondrial apoptotic pathway.

### Roc-A Promotes Mitophagy via the PINK1/Parkin Pathway in PC Cells

**Figure 4:**
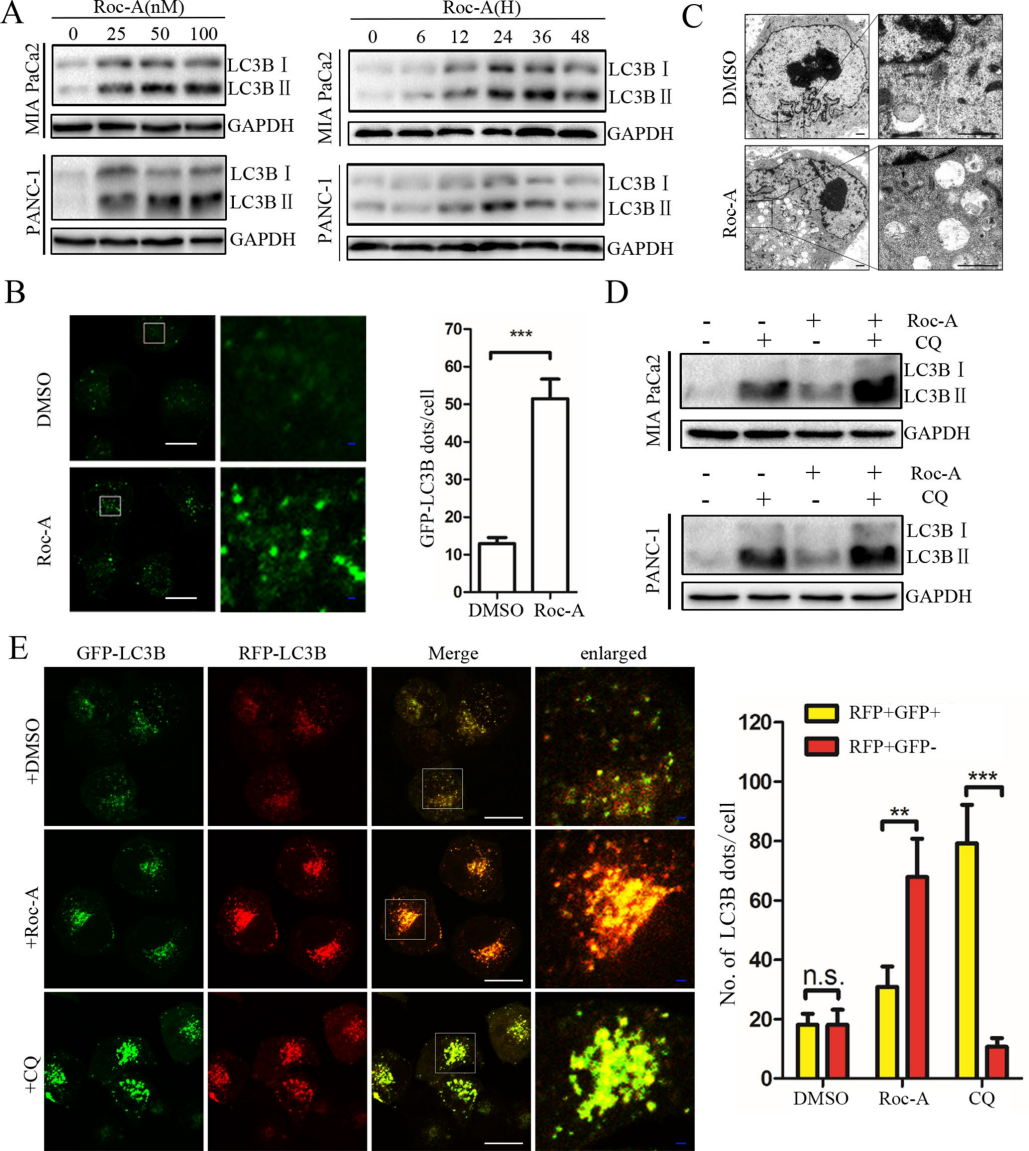
Roc-A promotes autophagy in PC cells. **(A)** Western blotting analyzed protein expression levels of LC3B II after cells were treated with Roc-A at the indicated concentration for 24 h or indicated duration in 50 nM. **(B)** Representative confocal images are shown. Cells transfected with GFP-LC3B were incubated with Roc-A (50 nM) for 24 h. LC3B dots quantified over 20 cells were included in two group. ***p < 0.001. Scale bar: 20 (white) and 5 µm (blue). **(C)** Representative images of autophagosomes/autolysosomes were detected by transmission electron microscopy. Scale bar: 1 µm. **(D)** Cells were incubated with Roc-A (50 nM) in the presence or absence of CQ (10 µM). Cell lysates were used to analyze the expression level of LC3B II. **(E)** Using confocal microscope observed the change of both green and red fluorescence after PANC-1 cell transfected with GFP-mRFP-LC3B was treated with DMSO (<0.1%), Roc-A (50 nM), or CQ (10 µM) for 24 h. The numbers of autophagosome puncta and autolysosome puncta were calculated. n.s., no significance; **p < 0.01; ***p < 0.001. Scale bar: 20 (white) and 5 µm (blue).

Autophagy is critical for maintaining cellular homeostasis and functions by the removal of damaged organelles (Williams et al., 2017). Although the regulatory machinery of the autophagic pathway has been well characterized, accurate modulation of this pathway remains complex and largely unclear in the context of clinical translatability for improved cancer therapies (Bhat et al., 2018). Thus, to investigate whether Roc-A induced cell autophagy in PC, we analyzed endogenous LC3B expression by western blotting and found that Roc-A induced LC3B-II expression in dose- and time-dependent manner in PC cells (**Figure 4A**). To monitor whether Roc-A induced autophagosome formation, we used a lentiviral vector encoding GFP-LC3B fusion protein, and we found that the number of GFP-LC3B puncta significantly increased in Roc-A-treated cells compared with the control group (**Figure 4B**). TEM also revealed that the number of autophagosomes has a significant increase in Roc-A-treated cells (**Figure 4C**). These results indicate that Roc-A causes autophagosome accumulation. The accumulation of autophagosomes can occur by either the blockage of autophagosome–lysosome formation or autophagy activation (He et al., 2018). Thus, CQ, which was a lysosome inhibitor, was used to examine the action of inhibiting the lysosomal turnover. As shown in **Figure 4D**, CQ could apparently increase Roc-A-induced LC3B-II accumulation in PC cells, suggesting that Roc-A led to LC3B-II accumulation because of autophagy activation. Next, we used a tandem GFP-mRFP-LC3B lentiviral to measure autophagic flux and identify autophagosomes and autolysosomes; GFP is sensitive to acidic lysosomal environment and easily quenched, while mRFP is resistant to acidic environments (Kawai et al., 2014). Our results showed that CQ increased yellow puncta without accompanying apparent increase of red puncta in PANC-1 (**Figure 4E**). On the contrary, the red fluorescent autolysosomes were obviously increased in Roc-A-treated cells. These results demonstrated that Roc-A could induce autophagy in PC.

**Figure 5:**
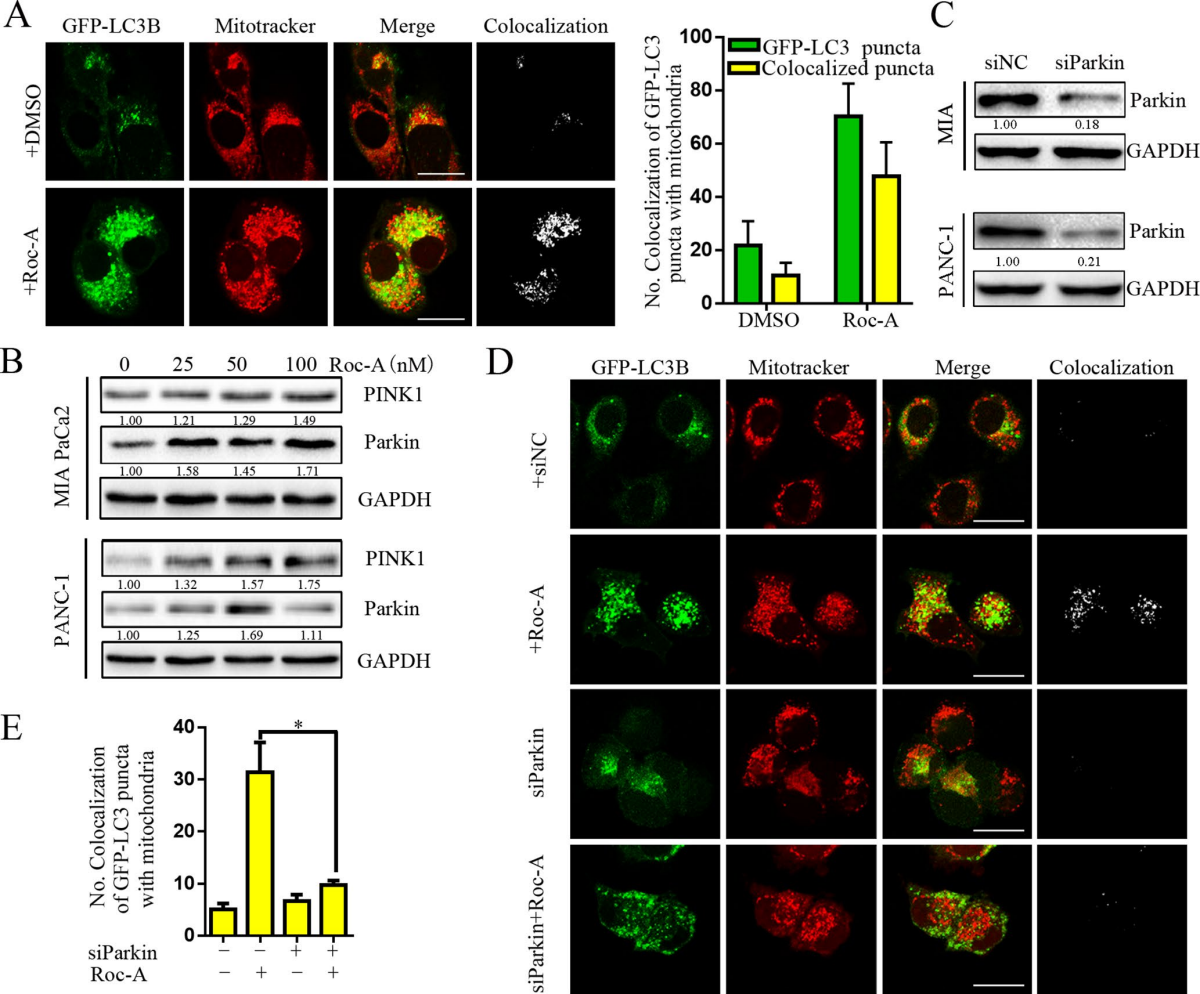
Roc-A promotes mitophagy via PINK1/Parkin pathway in PC cells. **(A)** Image of colocalization of GFP-LC3 puncta and MitoTracker Red is shown. Scale bar: 25 µm. **(B)** Western blotting analyzed the protein level of Parkin and PINK1. **(C)** Western blotting analyzed the protein level of Parkin after cells were transfected with siRNA against Parkin. **(D–E)** PANC-1 transfected with GFP-LC3B were incubated with or without Roc-A (50 nM) for 24 h and stained with MitoTracker Red. Confocal microscope was used to observe and photograph images. The numbers of autophagosome puncta and mitochondria puncta were calculated. *p < 0.05.

There is an increasing evidence that manifest that mitophagy plays a critical role in maintaining proper cellular function (Pickles et al., 2018). We thus examined whether Roc-A induced mitophagy. We first used GFP-LC3B and MitoTracker to separately mark the autophagosome and mitochondria, respectively, and analyzed subcellular localization of both. We observed that colocalization of autophagosomes and mitochondria, which was reflected by yellow fluorescent intensity, was significantly increased after Roc-A treatment compared with the control group (**Figure 5A**). Some studies have borne out that the PINK1/Parkin pathway, BNIP3L, and FUNDC1 are activated to mediate mitophagy and act as a sensor for mitochondrial quality (Rub et al., 2017). We found that Roc-A increased the expression levels of PINK1 and Parkin with the increase of dose (**Figure 5B**), while BNIP3L and FUNDC1 remained almost unchanged (**Supplement Figure 3**). To confirm our results, we used Parkin-specific siRNA and first confirmed that Parkin-specific siRNA effectively silenced Parkin protein levels in MIA and PANC-1 cells (**Figure 5C**). Parkin knockdown strikingly reduced the number of colocalized GFP-LC3B puncta with mitochondria in response to Roc-A compared with control siRNA (**Figure 5D**). Meanwhile, we silenced PINK1 protein levels by PINK1-specific siRNA and came to the same conclusion (**Supplement 4**). Taken together, these results indicate that Roc-A promotes mitophagy by the PINK1/Parkin pathway in PC cells.

### Inhibition of Autophagy/Mitophagy Augments Roc-A-Induced Apoptosis in PC Cells

**Figure 6:**
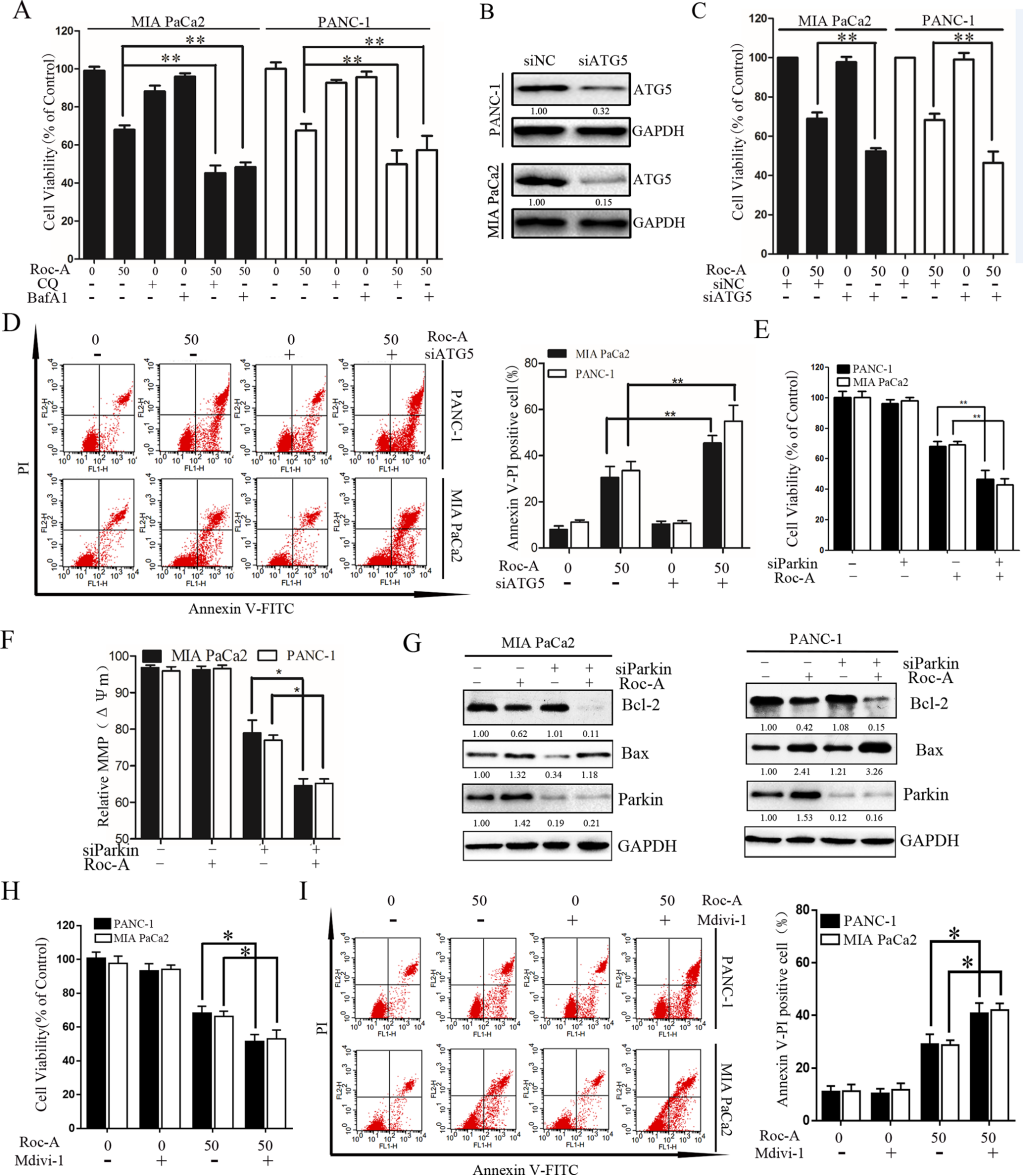
Inhibition of autophagy/mitophagy augments Roc-A-induced apoptosis in PC cells. **(A)** Cell viability was measured by CCK-8 assay after cells were treated with Roc-A (50 nM) in the presence or absence of CQ (10 µM) or Baf-A1 (10 µM) for 24 h. Data represented as means ± SD of three separate experiments. **p < 0.01. **(B)** Western blotting analyzed the protein level of ATG5 after cells were transfected with siRNA against ATG5. **(C)** Cell viability were analyzed by CCK-8 assay. Data represented as means ± SD of three separate experiments. **p < 0.01. **(D)** Analyzed apoptosis cell by flow cytometry. Data represented as means ± SD of three separate experiments. **p < 0.01. **(E**–**G)** Cells were transfected with siRNA against Parkin in the absence or presence with Roc-A (50 nM), and CCK-8 assay measured cell viability, JC-1 staining analyzed MMP (쉙*Δψm*), and western blotting analyzed the protein level of Bcl-2 and Bax. **(H)** Cell viability was measured by CCK-8 assay. *p < 0.05. **(I)** Flow cytometry detected apoptotic cells. Cells were incubated with Roc-A (50 nM) and Mdivi-1 (10 µM). Data represented as means ± SD of three separate experiments. *p < 0.05; **p < 0.01.

To determine the biological role of Roc-A-induced autophagy in PC, two lysosomal inhibitors, CQ and Baf-A1, were used to block lysosomal function. As shown in **Figure 6A**, Roc-A in combination with CQ or Baf-A1 resulted in significantly inhibited growth compared with Roc-A alone in MIA and PANC-1 cells. To eliminate the nonspecific effects of CQ or Baf-A1, we blocked autophagy using siRNA targeting ATG5 and analyzed the relationship between apoptosis and autophagy. As shown in **Figures 6B and C**, ATG5-specific siRNA effectively reduced ATG5 protein level and markedly increased Roc-A-induced proliferation inhibition in PC cells. ATG5 knockdown also increased Roc-A-induced cell apoptosis than did control siRNA (**Figure 6D**). We also found that cell viability and *Δψm* were further decreased and that cell apoptosis was increased in cells transfected with Parkin siRNA and treated with Roc-A than Roc-A treatment alone (**Figures 6E, F**). Moreover, we found further increased Bax levels and decreased Bcl-2 levels in cells transfected with Parkin siRNA and treated with Roc-A than Roc-A treatment alone (**Figure 6G**). Next, we blocked mitophagy selectively by using Mdivi-1 to check whether Roc-A-induced apoptosis and tumor growth delay were affected. Our results showed that Mdivi-1 combined with Roc-A resulted in significantly inhibited growth than did Roc-A alone and increased Roc-A-induced apoptosis (**Figures 6H, I**). Together, these data suggested that inhibition of autophagy/mitophagy can augment Roc-A-induced apoptosis in PC cells and that Roc-A-induced autophagy/mitophagy plays a protective role in PC cell apoptosis.

### Roc-A-Induced Autophagy/Mitophagy and Apoptosis Involves ROS Production

**Figure 7:**
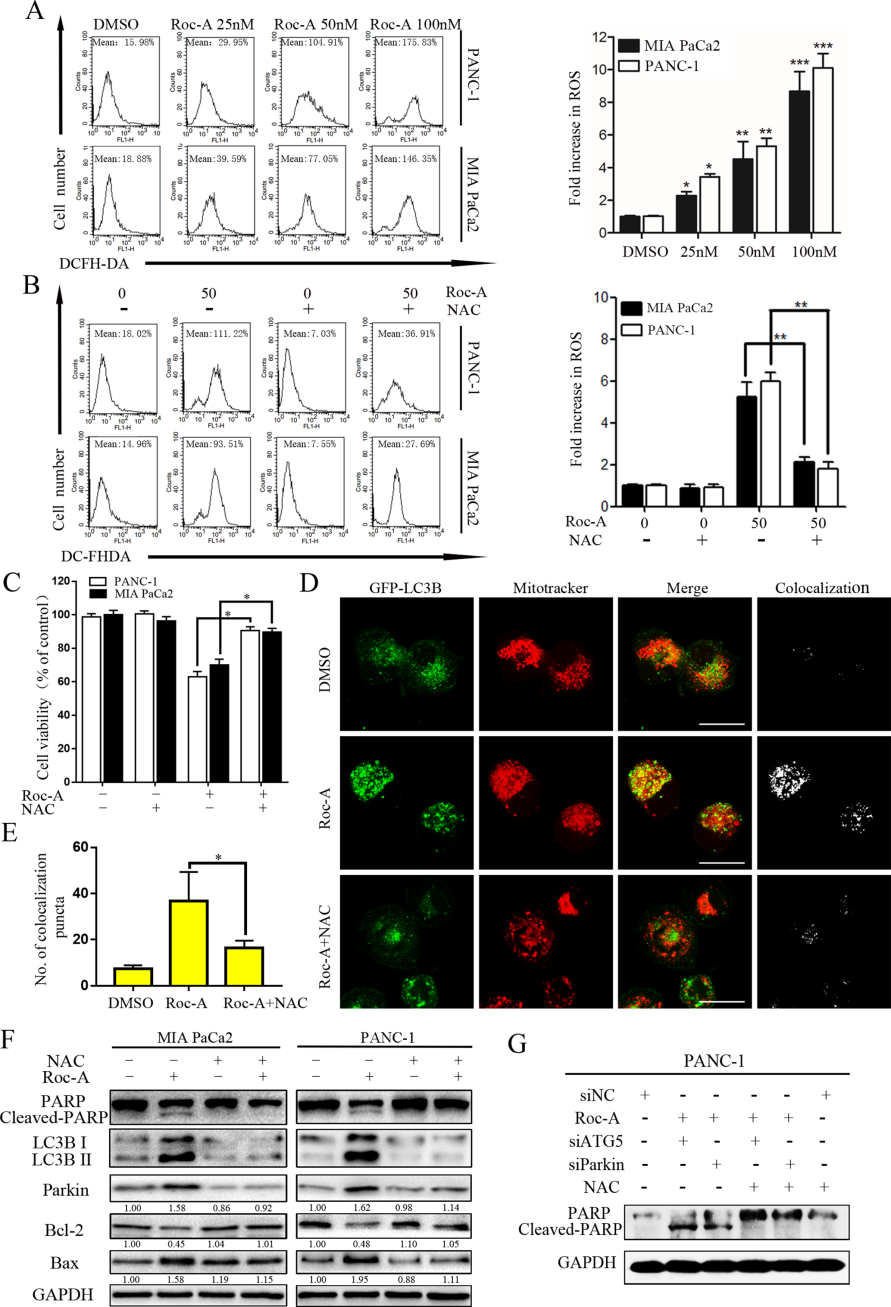
Roc-A-induced mitophagy and apoptosis were involved in ROS production. **(A, B)** Cells were treated with Roc-A with or without NAC (5 mM) for 24 h and examined ROS production by flow cytometry. Each bar represents means ± SD of three separate experiments. *p < 0.05; **p < 0.01; ***p < 0.001. **(C)** Cell viability was measured by CCK-8 assay after cells were treated with Roc-A (50 nM) with or without of NAC (5 mM) for 24 h. Data represented as means ± SD of three separate experiments. **p < 0.01. **(D, E)** PANC-1 transfected with GFP-LC3B were incubated with or without Roc-A (50 nM) for 24 h and stained with MitoTracker Red. Confocal microscope was used to observe and photograph images. The numbers of yellow (colocalization of autophagosomes and mitochondria) puncta are shown in the panel. *p < 0.05. **(F)** Western blotting analyzed the protein level after cells were treated with Roc-A with or without of NAC (5 mM) for 24 h. **(G)** Western blotting analyzed PARP level after cells were treated with Roc-A, NAC together, with siATG5 or siParkin.

Endogenous ROS is a by-product of normal mitochondrial metabolism mainly formed in the mitochondrial respiratory chain. Moderate formation and release of ROS play a critical role in regulating biological functions and cell homeostasis, while excessive production and release of ROS can induce cell death by multiple mechanisms (Zou et al., 2017). To evaluate whether Roc-A-induced cell apoptosis is involved ROS production, we examined ROS generation using the fluorescent probe DCFH-DA, and the level of ROS increased in response to Roc-A (**Figure 7A**). We next researched whether excessive release of ROS related with Roc-A induced cell viability inhibition. Cells were pretreated with the ROS scavenger NAC; our results demonstrated that NAC can apparently abrogate Roc-A-induced ROS production (**Figure 7B**) and strikingly rescue Roc-A-induced cell growth inhibition (**Figure 7C**). Next, to validate the effect of NAC on Roc-A-induced mitophagy, we used GFP-LC3B and MitoTracker to separately mark the autophagosome and mitochondria, and we analyzed subcellular localization of both. We observed that colocalization of autophagosomes and mitochondria, which was reflected by yellow fluorescent intensity, was significantly decreased after NAC combined with Roc-A treatment compared with Roc-A group alone (**Figures 7D, E**). Additionally, we used NAC together with siATG5 or siParkin to check whether Roc-A-induced apoptosis is affected or not. Our results show that NAC can reverse the level of cleaved PARP, LC3B-II, Bax, and Bcl-2 (**Figure 7F**). Next, we use NAC together with siATG5 and siParkin to check whether Roc-A-induced apoptosis is affected or not. We found that cell viability was significantly increased (**Supplement 5**) and apoptosis is obviously decreased (**Figure 7G**) after cells were incubated with NAC. Together, these results demonstrated that Roc-A-induced PC cell apoptosis and autophagy/mitophagy were mediated by ROS.

## Discussion

In our research, we clarified the action and underlying mechanisms of Roc-A on apoptosis and mitophagy in PC cells. Our data demonstrated that Roc-A induces mitophagy by the PINK1/Parkin signal pathway and provokes PC cell apoptosis by generation of intracellular ROS, loss of the *Δψm*, and release of cytochrome *c*. We also found that inhibition of autophagy/mitophagy can augment Roc-A-induced cell apoptosis in PC cells. To the best of our knowledge, our research is the first to elucidate this novel mechanism for Roc-A-mediated PC cell death.

Cell apoptosis is regulated by numerous signal pathways, for instance, caspase family and Bcl-2 protein family. Caspases are a family of cysteine proteases and major regulators of cell apoptosis (Porter and Jänicke, 1999). The Bcl-2 family protein is critical in regulating and controlling intrinsic apoptosis pathway and is involved in controlling the *Δψm* (Gross, 2016). Our results indicated that Roc-A-induced PC cell death involved a mitochondria apoptosis pathway by loss of the *Δψm*, release of cytochrome *c*, and activation of caspase-3 and PARP. Moreover, Roc-A treatment upregulated the expression Bax and downregulated the expressions of Bcl-XL and Bcl-2. Consistent with our results, previous studies showed that Roc-A induces depolarization of the *Δψm* and triggers caspase-mediated apoptosis through regulation of Bcl-2 family members in colorectal cancer and leukemia cells (Hausott et al., 2004, Zhu et al., 2007).

Autophagy is a self-eating cytoplasmic components process; cytoplasmic components and organelles were packaged to form autophagosomes and degraded in lysosomes (Codogno et al., 2011). The therapeutic effect of autophagy in cancer has been controversial (Levy et al., 2017). Some studies showed that autophagy can promote tumor cell survival by the clearance of damaged or superfluous organelles (El-Hattab et al., 2018). Mitophagy is a selective autophagy of mitochondria, which plays a critical role in mitochondrial quality control mechanism. Mitophagy can maintain and promote cell survival by eliminating damaged and dysfunctional mitochondria (Ashrafi and Schwarz, 2013). In our research, we found that Roc-A resulted in accumulation of autophagosomes, which led to accelerated autophagosome synthesis in PC cells. We further demonstrated that Roc-A induced mitophagy by the PINK1/Parkin signal pathway. To evaluate the role of Roc-A-induced autophagy/mitophagy in PC cell apoptosis, we measured cell viability in the presence of Roc-A with autophagy inhibitors or ATG5 or Parkin knockdown. Notably, inhibition of autophagy/mitophagy increased Roc-A-induced mitochondrial apoptosis, implying that Roc-A-induced autophagy/mitophagy was a protection measurement. Similar to our findings, previous studies have also reported that PINK1/Parkin-mediated mitophagy plays a protective role in a variety of tumors. For instance, one report showed that Matrine induces liver cancer cell apoptosis by inhibiting the PINK1/Parkin pathway and mitophagy (Wei et al., 2018). Some researchers also demonstrated that TNFα activates Parkin-dependent mitophagy to block mitochondrial apoptosis in gastric cancer cells (Yan et al., 2018), while a very recent study demonstrated that Roc-A enhanced natural killer (NK) cell-mediated lysis through inhibition of autophagy. Interestingly, it is shown that Roc-A blocks the protein translation of ULK1 (Yao et al., 2018). We think that Roc-A-induced autophagy/mitophagy depends on cell type and tissue specificity.

ROS plays a vital role in the regulation of cell survival (Nogueira and Hay, 2013). Moderate levels of ROS can maintain cellular and tissue homeostasis and promote cell survival; however, when intracellular ROS is released in large quantities, ROS can induce cell death (Trachootham et al., 2008). Our results show that Roc-A can induce ROS production in PC cells. Furthermore, pretreatment with the ROS scavenger NAC significantly reversed Roc-A-induced mitochondrial dysfunction, cell apoptosis, and autophagy/mitophagy, indicating that increase of intracellular ROS levels may be the major mechanism of cell death induction by Roc-A. Consistent with our results, numerous other natural products have also been reported to induce ROS generation to produce a killing effect on cancer cells (Cho et al., 2018, Kang et al., 2018)

Taken together, our results suggest that Roc-A induced cell death and tumor suppression by induction of mitochondria dysfunction and ROS production in PC. Furthermore, we found that Roc-A induces PINK1/Parkin-associated mitophagy, which plays a protective role in Roc-A-induced PC cell apoptosis. Our study proposes a novel explanation for the mechanism of action for Roc-A, indicating Roc-A as a prospective therapeutic drug against PC and emphasizing that the combination inhibition of autophagy/mitophagy is a probably promising therapeutic method in tumor suppression.

## Data Availabilty

All datasets generated for this study are included in the manuscript and/or the **Supplementary Files**.

## Ethics Statement

Animal experiments were approved by the Institutional Animal Care and Treatment Committee of Huazhong University of Science and Technology and performed in accordance with the Association for Assessment and Accreditation of Laboratory Animal Care guidelines.

## Author Contributions

Conceptualization was done by CZ, RH, and XL. Data curation was done by CZ. Formal analysis was done by HC. Funding acquisition was done by FZ, MW, and RQ. Investigation was done by CZ and RH. Methodology was done by RH, XL, and RQ. Project administration was done by XL and RQ. FZ and YL were in charge of software. MS was in charge of supervision and visualization. Writing of the original draft was done by CZ. Writing, including review and editing, was done by XL and RQ.

## Funding

This work was supported by the NSFC (No. 81772950, No. 81502633, No. 81874205, and No. 81773160). HuBei Natural Science Foundation (2017CFB467), Wuhan applied basic research project (2016060101010070), and Tongji Hospital Science Fund for Distinguished Young Scholars (2016YQ08).

## Conflict of Interest Statement

The authors declare that the research was conducted in the absence of any commercial or financial relationships that could be construed as a potential conflict of interest.
