# Supplementary material for: PINK1/Parkin-Mediated Mitophagy Regulation by Reactive Oxygen Species Alleviates Rocaglamide A-Induced Apoptosis in Pancreatic Cancer Cells
Source: Front Pharmacol. 2019 Sep 3;10:968. doi: 10.3389/fphar.2019.00968 (PMC6735223; doi:10.3389/fphar.2019.00968)
Supplement: Supplementary file 1 [file DataSheet_1.docx]

PINK1/Parkin-mediated mitophagy regulation by reactive oxygen species alleviates Rocaglamide A-induced apoptosis in pancreatic cancer cells

Chunle Zhao ^1, *^ , Ruizhi He ^1,*^ , Ming Shen ^1,*^ , Feng Zhu ^1^, Min Wang ^1^, Yuhui Liu ^1^, Hua Chen ^1^, Xu Li^1,#^, Renyi Qin^1,#^

^1^Laboratory of Biliary-Pancreatic Surgery, Department of Biliary-Pancreatic Surgery, Affiliated Tongji Hospital, Tongji Medical College, Huazhong University of Science and Technology, Wuhan, Hubei, China

*****These authors equally contributed to this research

^#^Corresponding author: Xu Li([2013tj0574@hust.edu.cn](mailto:2013tj0574@hust.edu.cn)); Renyi Qin([ryqin@tjh.tjmu.edu.cn](mailto:ryqin@tjh.tjmu.edu.cn))


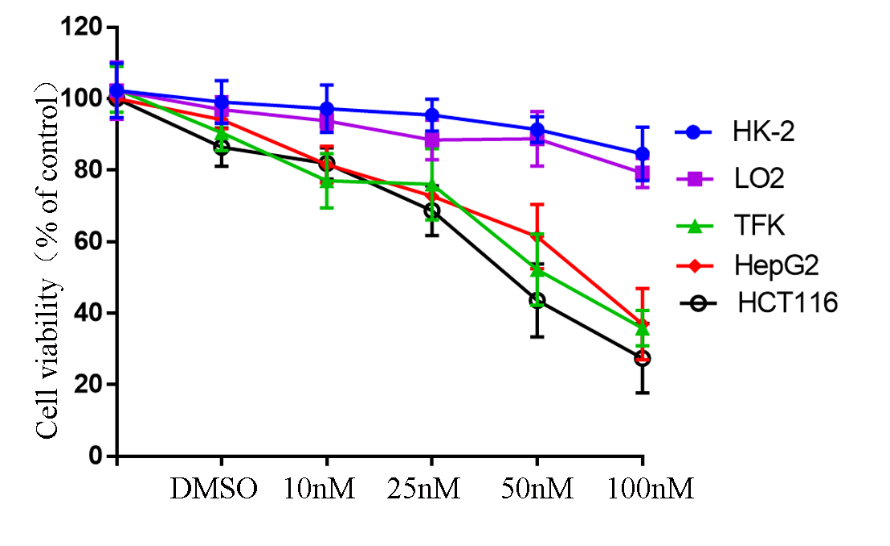


**Supplementary Figure 1. Roc-A inhibits growth of other gastrointestinal tumor cells growth in vitro.**

Cell viability was measured by CCK-8. Data are presented as mean ± SD from three independent experiments. HK-2 (human renal tubular epithelial cell); LO2 (human live cell); TFK-1 (human cholangiocarcinoma cell); HepG2 (human hepatoma carcinoma cell); HCT-116 (human colon cancer cell).


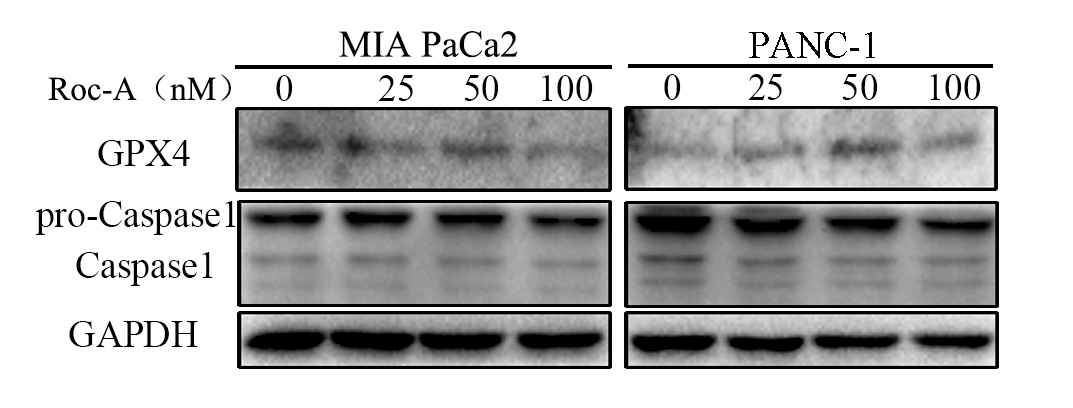


**Supplementary Figure 2. Roc-A induces cell apoptosis is not involved in ferroptosis and pyroptosis.**

Western blot analyzed the levels GPX4 and Caspase-1. Cells were treated with Roc-A at the indicated concentration for 24 hours, and extracted the supernatant for western blot.


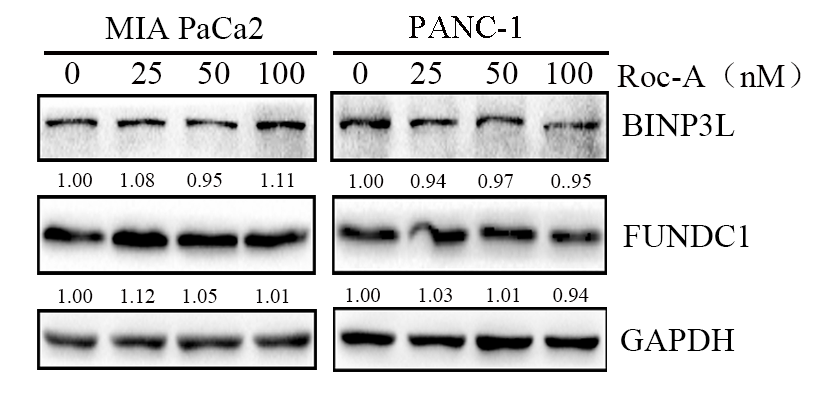


**Supplementary Figure 3. Roc-A promotes mitophagy is not involved in BNIP3L and FUNDC1 in PC cells.**

Western blot analyzed the protein level of BNIP3L and FUNDC1.

**
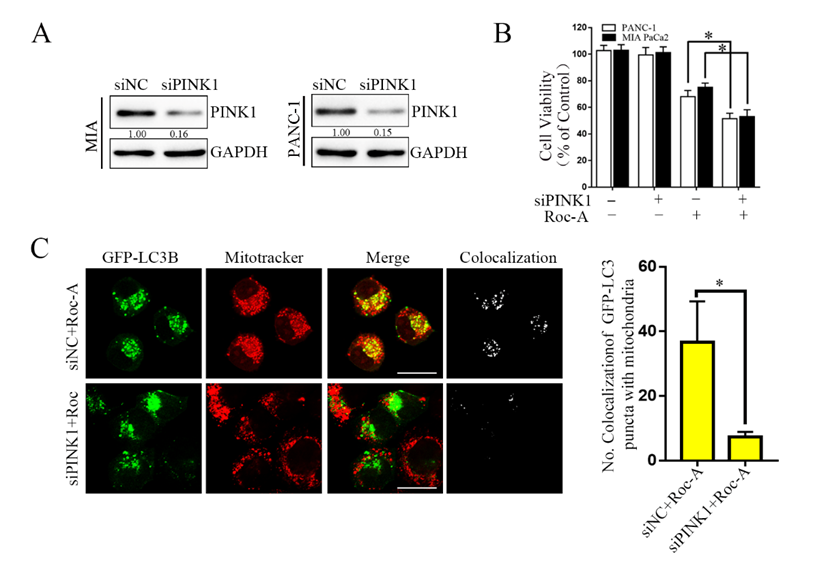
**

**Supplementary Figure 4. Silenced PINK1 can also reduce Roc-A-induced cell viability and mitophagy.**

Western blotting analyzed the protein level of PINK1 after cells were transfected with siRNA against PINK1. B. CCK-8 assay measured cell viability after cells were transfected with siRNA against PINK1 in the absence or presence with Roc-A (50 nM). Data are presented as mean ± SD from three independent experiments. The asterisks indicate a statistically significant effect of treatment. *, p <. 05. C. PANC-1 transfected with GFP-LC3B were incubated with or without Roc-A (50 nM) for 24 hours and stained with MitoTracker Red. Confocal microscope was used to observe and photograph images.

**
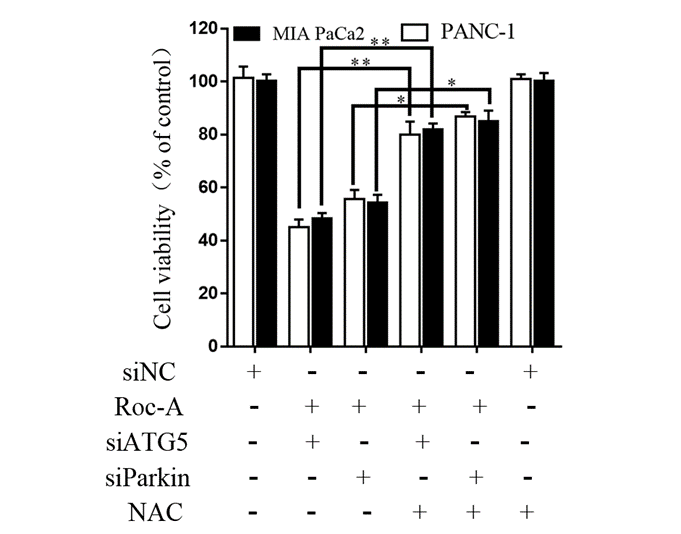
**

**Supplementary Figure 5.** NAC can decrease Roc-A together with siATG5 and siParkin induced cell death.

CCK-8 assay measured cell viability after cells were treated with Roc-A(50nM) together with siATG5 and siParkin or NAC (5 mM) for 24 hours. Data are presented as mean ± SD from three independent experiments. The asterisks indicate a statistically significant effect of treatment. *, p <. 05; **, p <. 01.
